# Rapid Nitration of Adipocyte Phosphoenolpyruvate Carboxykinase by Leptin Reduces Glyceroneogenesis and Induces Fatty Acid Release

**DOI:** 10.1371/journal.pone.0040650

**Published:** 2012-07-11

**Authors:** Anne-Marie Jaubert, Graziella Penot, Fatoumata Niang, Sylvie Durant, Claude Forest

**Affiliations:** 1 Institut National de la Santé et de la Recherche Médicale UMR-S 747; Université Paris Descartes, Pharmacologie Toxicologie et Signalisation Cellulaire, Paris, France; 2 Département de Biochimie et de Biologie Moléculaire, Faculté de Médecine Paris-Ile de France-Ouest; Université de Versailles Saint-Quentin en Yvelines, Versailles, France; Karolinska Insitutet, Sweden

## Abstract

Fatty acid (FA) release from white adipose tissue (WAT) is the result of the balance between triglyceride breakdown and FA re-esterification. The latter relies on the induction of cytosolic phosphoenolpyruvate carboxykinase (PEPCK-C), the key enzyme for glyceroneogenesis. We previously demonstrated that long-term (18 h) leptin treatment of rat epididymal WAT explants reduced glyceroneogenesis through nitric oxide (NO)-induced decrease in PEPCK-C expression. We investigated the effect of a short-term leptin treatment (2 h) on PEPCK-C expression and glyceroneogenesis in relation to NO production. We demonstrate that in WAT explants, leptin-induced NO synthase III (NOS III) phosphorylation was associated with reduced PEPCK-C level and glyceroneogenesis, leading to FA release, while PEPCK-C gene expression remained unaffected. These effects were absent in WAT explants from leptin receptor-deficient Zucker rat. Immunoprecipitation and western blot experiments showed that the leptin-induced decrease in PEPCK-C level was correlated with an increase in PEPCK-C nitration. All these effects were abolished by the NOS inhibitor Nω-nitro-L-arginine methyl ester and mimicked by the NO donor S-nitroso-N-acetyl-DL penicillamine. We propose a mechanism in which leptin activates NOS III and induces NO that nitrates PEPCK-C to reduce its level and glyceroneogenesis, therefore limiting FA re-esterification in WAT.

## Introduction

The sustained high level of plasma fatty acids (FA) can concur to the onset of insulin resistance potentially resulting in type 2 Diabetes, a situation frequently encountered in obese individuals [1], [2]. White adipose tissue (WAT) is the FA-producing tissue. A large series of studies has been focused on the regulation of lipolysis, i.e. FA release from WAT [3]–[5]. FA output is the result of triglyceride breakdown, β-oxidation and FA re-esterification [6], [7]. The latter requires glycerol-3P synthesis from lactate, pyruvate, or certain amino acids, as the endpoint of a pathway named glyceroneogenesis [8], [9]. The cytosolic isoform of phosphoenolpyruvate carboxykinase (PEPCK-C) is the key enzyme of this metabolic pathway [10]. Several nutrients and hormones modulate glyceroneogenesis by means of alterations in PEPCK-C gene (*Pck1*) regulation, particularly at the transcriptional level, that alters PEPCK-C synthesis [11], [12].

PEPCK-C is also the key gluconeogenic enzyme in the liver and most studies were focused on this organ and on liver-derived cells - hepatocytes and hepatoma cells - for years [10], [12]. Up to recently, the common thinking was that the balance between synthesis and degradation determined the total cellular PEPCK-C activity because no allosteric modifier of this enzyme was described and no factor that regulated its rate of degradation was known [13], [14]. However, more recent data showed that the acetylation of hepatic PEPCK-C resulted in the rapid decrease in the level of this enzyme and gave evidence that such a post-translational modification was an adequate way for glucose to rapidly repress gluconeogenesis [15], [16]. This type of regulation appears therefore to be an acute means for adapting liver glucose production to the needs.

We recently demonstrated that a long-term treatment (18 h) with leptin reduced glyceroneogenesis, PEPCK-C mRNA and PEPCK-C concentration in rat WAT explants [17]. We also showed that this process depended on nitric-oxide (NO) production [17]. Such a long-term action of leptin resulted also in a decrease in lipolysis whereas a treatment for 2 h stimulated lipolysis [17]–[19]. A short-term lipolytic effect of NO was also observed when NO donors were used [20].

NO has been proposed as a potential mediator of metabolic disturbances linked to obesity [21], [22]. Three NO synthase (NOS) isoforms regulate NO production: endothelial (eNOS or NOS III), neuronal (nNOS or NOS I) and the inducible (iNOS or NOS II) NOS. In most species and tissues, NOS II is expressed at very low levels under normal conditions. Upon induction of expression of its gene, NOS II generates NO in larger amounts and much later than the NO produced in response to NOS III activation [23]. NOS II and III are expressed in rat adipocytes [24]. We showed that leptin stimulation acutely leads to the phosphorylation of NOS III with a resulting increase in NO production in isolated adipocytes [25]. In contrast, other cytokines, like tumor necrosis factor alpha or interferon gamma (IFN-γ), affected adipocyte metabolism via the induction of NOS II expression, a process that took several hours to be achieved [23], [26], [27]. A large series of studies demonstrated that a rise in NO levels could cause protein modifications like nitrosylation and nitration that could both result in an accelerated degradation of the protein [27]–[29]. Furthermore, tyrosine nitration could alter protein functions and be associated to acute and chronic disease pathologies [30]–[32].

The aim of the present study was to examine the role of a short-term (2 h) treatment with leptin on glyceroneogenesis and PEPCK-C in rat WAT explants in relation with NOS III activation. We show that leptin, but not IFN-γ, activates NOS III, inducing NO and the nitration of PEPCK-C, therefore reducing PEPCK-C amount without affecting the expression of its gene. This sequence of events results in a decrease in glyceroneogenesis and FA re-esterification leading to an increase in FA release.

**Table 1:** Rat probes used in real time PCR.

Classification	Gene symbol	Gene name	Context sequence
Endogeneous control	*Rnr 1*	Eukaryotic 18S ribosomal RNA	^5′^ TCCCCCAACTTCTTAGAGG ^3′^ ^3′^ CTTATGACCCGCACTTACTG ^5′^
Lipid metabolism	*Pck 1*	Phosphoenolpyruvate carboxykinase cytosolic	^5′^TGATGAACCCCACCCTC ^3′^ ^3′^ TCCCAGTAAACACCCCC ^5′^
NO synthesis	*Nos 2*	Nitric oxide synthase II	^5′^TGAGGGGACTGGACTTTTAG ^3′^ ^3′^ CTGTGACTTTGTGCTTCTGC ^5′^
NO synthesis	*Nos 3*	Nitric oxide synthase III	^5′^GCATACCCGCACTTCTG ^3′^ ^3′^CCTTGGCATCTTCTCCC ^5′^

## Materials and Methods

### Materials

Dulbecco’s Modified Eagle Medium (DMEM), penicillin, streptomycin and TRizol® Reagent (Total RNA Isolation Reagent) were from Invitrogen Life Technologies (Cergy-Pontoise, France). SV total RNA Isolation System kit was purchased from Promega (Charbonnières-les-bains, France), High Capacity cDNA Archive kit was from Applied Biosystems (Courtaboeuf, France) and Absolute qPCR SYBR green Rox mix from Thermo Scientific (Villebon-sur-Yvette, France). Leptin and INF-γ were from Peprotech (Rocky Hill, NJ, USA). [1-^14^C] pyruvic acid, sodium salt (1 mCi/L), Hybond-N^+^ membranes and Hyperfilm™ ECL were from Amersham Biosciences (Buckinghamshire, UK). Free Fatty Acids Half Micro Test was from Roche (Manheim, Germany) and glycerol (glycerol UV-method) from R-Biopharm (Saint Didier au Mont D’Or, France). S-Nitroso-N-Acetyl-DL Penicillamine (SNAP) was purchased from Cayman (SPI BIO, France). α-Cyano-(3,4-dihydroxy)-N-benzylcinnamide (AG490) was from Calbiochem (San-Diego, CA, USA). Fetal bovine serum, essentially FA-free bovine serum albumin (BSA), Nω-nitro-L-arginine methyl ester (L-NAME), sodium pyruvate and all other products were purchased from Sigma (L’isle d’Abeau Chesnes, France). For Western blot analysis, sodium dodecyl sulfate (SDS) – polyacrylamide gel electrophoresis (PAGE) was performed using a SDS-MOPS running buffer and a Novex 4–12% Bis-Tris gel from Invitrogen Life Technologies (Carlsbad, CA, USA). PEPCK-C antibody was a gift from Pr E. Beale (TTUHC, USA) [33] and the rabbit polyclonal anti-nitrotyrosine antibody was from US Biological, Swampscott, MA, USA. Anti-phosphoserine^1179^ NOS III antibody was purchased from Cell Signaling Technology (Beverley, MA, USA). Polyclonal anti-NOS III was from Transduction Laboratories (San-Diego, CA, USA). Beta-actin antibody was from Santa-Cruz Biotechnology, CA, USA.

### Animals

Male Sprague–Dawley (SD) rats obtained from Centre d’Elevage de Rats Janvier (Le Genest St Isle, France) at 8 weeks of age, were maintained at constant room temperature (24°C) on a 12 h light/dark cycle. Male Zucker fatty rats and their lean littermates at 8 weeks of age were purchased from Charles River laboratories (L’Arbresle, France). Rats were killed by decapitation and WAT from epididymal fat depots was carefully removed and rapidly used for fat pad preparation. All experimental protocols were approved by the Animal Use and Care Committee of the University.

### Culture of Explants and Assessment of FA Re-esterification

Epididymal fat pads were cut into 200 mg fragments in 1.5 ml of Krebs-Ringer Bicarbonate (KRB) buffer containing 2% fatty acid-free bovine serum albumin, 5 mM pyruvate and 20 µM [1-^14^C]-pyruvate (1 µCi/ml) as precursor of G3P. The isotopic dilution of radiolabeled substrates was kept constant at about 1∶250. Explants were then incubated in a humidified atmosphere of 10% CO_2_ at 37°C in the absence or presence of leptin (10 ng/ml) or INF-γ (50 ng/ml). To assess NO implication, we used the NO donor, SNAP (1 mmol/L) and the NO synthase inhibitor L-NAME (1 mmol/L). Two hours later, the incubation medium was collected for the estimation of lipolytic FA and glycerol. Simultaneously, the corresponding tissue fragments were frozen in liquid nitrogen before lipid extraction according to the method of Bligh and Dyer [34]. The subsequent [1-^14^C]-pyruvate incorporation into the lipid moiety was estimated by counting the radioactivity associated with this fraction and was used to appreciate the level of re-esterified FA during the lipolytic process.

### RNA Analysis, Reverse Transcription and Real-time PCR

Total RNA was extracted from the cell lines and from rat WAT by the method of Chomczynski and Sacchi [35], and Dnase-treated with the SV total RNA Isolation System kit.

For real-time RT-PCR analyses, 900 ng total RNA was first reverse transcribed using the High Capacity cDNA Archive kit. cDNAs were amplified in an ABI prism 7900 HT using the SYBR green fluorescence method and specific oligonucleotides.

Results were analysed with the SDS 2.1 real-time detection system software. Ribosomal 18S RNA (*Rnr1*) was used to normalise cDNA. Quantification of mRNA was carried out by comparison of the number of cycles required to reach reference and target threshold values (δ-δ Ct method). Sequences of the sense and antisense nucleotides corresponding to the different tested genes are given in Table 1.

**Figure 1:**
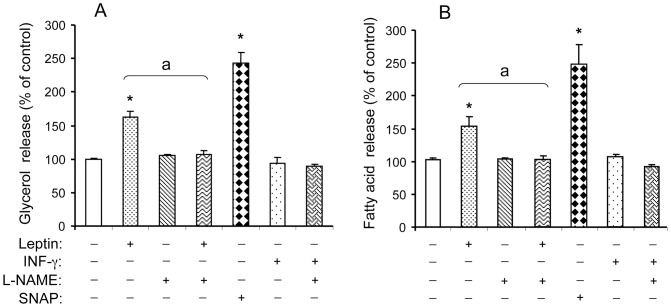
NO-dependent effects of leptin on glycerol (A) and FA (B) release from rat WAT explants. Explants were pre-treated or not with L-NAME (1 mmol/L) for 30 min, then exposed or not to either leptin (10 µg/L), SNAP (1 mmol/L) or IFN-γ (50 µg/L) for 2 h in KRB medium containing 2% BSA (lipolysis medium). Results are expressed as the percent of glycerol or FA relative to the corresponding untreated control. Crude values for control were 4.57±0.13 nmol.mg^−1^ tissue.2 h^−1^ for glycerol and 4.60±0.47 nmol.mg^−1^ tissue. 2 h^−1^ for FA. Each value represents the mean ± SEM, (n = 4) *, *P*<0.01 *vs*. control; ^a^ *P*<0.01 *vs.* leptin-treated explants.

**Figure 2:**
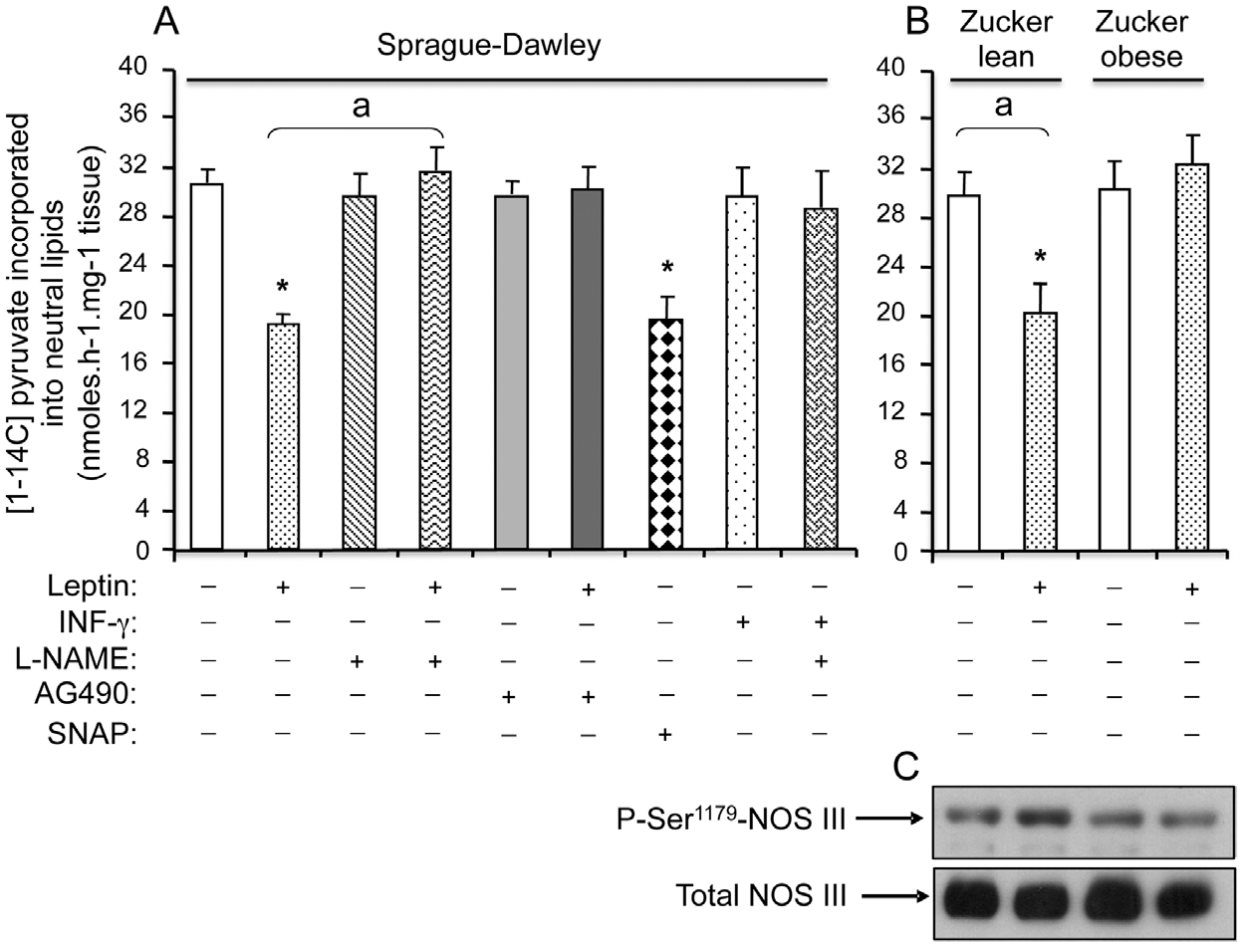
NO-dependent effects of leptin on glyceroneogenesis and NOS III phosphorylation in WAT explants from rats. Explants from SD (A) or Zucker rats (B, C) were pre-treated or not with L-NAME (1 mmol/L) or AG490 (10 µmol/L) for 30 min, then exposed or not to either leptin (10 µg/L), SNAP (1 mmol/L) or IFN-γ (50 µg/L) for 2 h in KRB medium containing 2% BSA and [1-^14^C]-pyruvate. Glyceroneogenic flux was measured by the [1-^14^C]-pyruvate incorporation into neutral lipids. Each value represents the mean ± SEM, (n = 4) *, *P*<0.01 *vs*. control; ^a^ *P*<0.01 *vs.* leptin-treated explants. (C) Representative autoradiogram of a western blot performed on WAT cytosolic proteins from Zucker rats reveals total NOS III and its Ser^1179^ phosphorylated form.

### Protein Analysis

Protein fraction was prepared from WAT explants, in PBS 1X buffer, containing 1% Nonidet P-40, 0.5% sodium deoxycholate, and 0.1% SDS to extract mitochondrial proteins. Protein concentrations were determined using the Bradford method with BSA as the standard [36]. Fractions (15 µg) were subjected to SDS-PAGE using a 10% resolving gel with a 6% stacking gel. Resolved proteins were transferred electrophoretically to nitrocellulose membranes, blocked for 1 h30 at 22°C with Tris-buffered saline (TBS) supplemented with 3% (w/v) BSA and 0.01% Tween and incubated overnight at 4°C with a 1/2500 dilution of the PEPCK-C antibody. The anti-NOS III and anti-actin antisera were used at a 1/1000 and a 1/500 dilution respectively. Nitrocellulose membranes were then washed with 1% Tween in TBS (three times for 5 min) and incubated with horseradish peroxidase–linked secondary antibody IgG (1/5000 dilution, in 1% [w/v] BSA in TBS with 0.05% Tween) for 1 h at 22°C. For PEPCK-C and NOS III detection, bound antibodies were visualized using ECL according to the manufacturer’s instructions. The blots were exposed to Hyperfilm, and the signals were quantified by scanning densitometry. For actin, the Odyssey method was used for detection, following the procedure described by the manufacturer (Li-COR).

### Analysis of the Nitrated Protein

The analysis of the nitrated PEPCK-C was determined as reported by Pilon et al. with slight modifications [27]. Two hundred µg of tissue lysate proteins were immunoprecipitated with 2.5 µg of anti PEPCK-C antibody coupled to protein A/G-magnetic beads (Bio Adembeads pAG, Ademtech) overnight at 4°C. The immune complex was washed three times in ice cold PBS buffer (pH 7.4) containing 0.05% Tween 20, and then resuspended in 40 µL SB buffer 1X (60 mmol/L Tris buffer pH = 6.8, 2% SDS, 0.1% bromophenol blue, 10% glycerol, 100 mmol/L DTT) and boiled for 3 min. Proteins were resolved on SDS-PAGE (4–12% gel) and processed for Odyssey western blot analysis with a 1/250 dilution of the anti-nitrotyrosine antibody.

### Image Quantification and Analysis of the Data

Quantitative results of Western blotting were obtained by densitometry in ImageJ software. The nonparametric Mann-Whitney *U* test for pairwise comparisons was applied due to the small number of experiments. Analyses were performed using the StatView 4.01 (Abacus Concepts, Berkeley, CA) statistical package. A value of *P*<0.05 was considered statistically significant.

**Figure 3:**
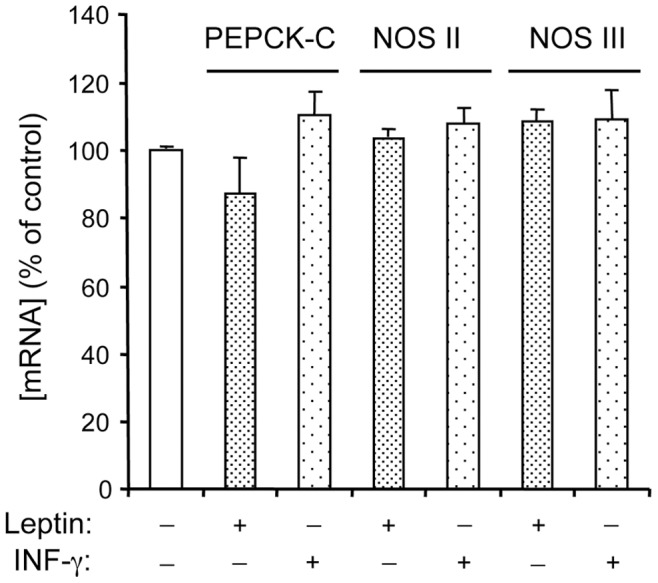
Effects of leptin and IFN-γ on PEPCK-C, NOS II and NOS III mRNA in rat WAT explants. Explants were treated or not with leptin (10 µg/L) or IFN-γ (50 µg/L) for 2 h. PEPCK-C, NOS II and NOS III mRNA levels were analysed by RT-qPCR. Results are normalized using 18S rRNA. Each value represents the mean ± SEM, (n = 4).

**Figure 4:**
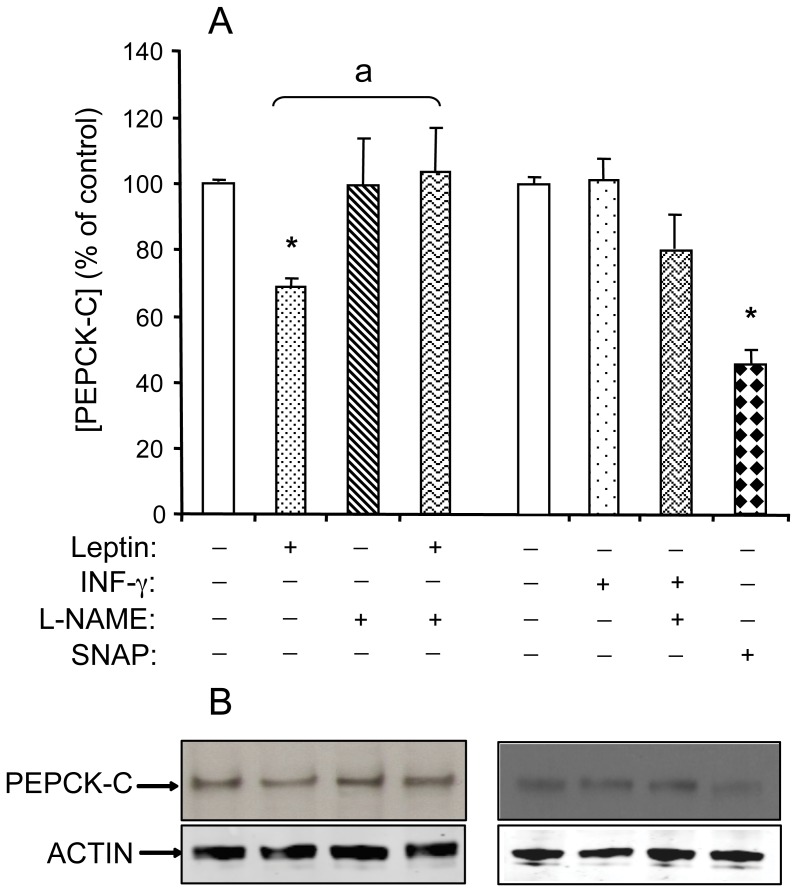
NO-dependent effects of leptin on PEPCK-C protein in WAT explants from rats. WAT explants were pre-treated or not with L-NAME (1 mmol/L) for 30 min, then exposed or not to either leptin (10 µg/L), SNAP (1 mmol/L) or INF-γ (50 µg/L) for 2 h in KRB medium containing 2% BSA. PEPCK-C and β-actin proteins were revealed by western blotting performed on cytosolic proteins. (A) Densitometry scanning in ImageJ software. Each value represents the mean ± SEM (n = 4) *, *P*<0.01 *vs*. control;^ a^ *P*<0.01 *vs.* leptin-treated explants. (B) Representative autoradiograms.

## Results

### Effect of Leptin and Interferon-gamma on Lipolysis and Glyceroneogenesis: Reversion by L-NAME

The lipolytic effects of leptin were studied in epididymal WAT explants from SD rats that were exposed or not to 10 ng/mL leptin or 50 ng/mL INF-γ for 2 h. Leptin induced a significant increase in glycerol and FA released in the incubation medium while INF-γ had no effect (Figure 1A, B). The NOS inhibitor L-NAME (1 mmol/L) alone was inefficient but it suppressed leptin effect whereas the NO donor SNAP (1 mmol/L) strongly stimulated both glycerol and NEFA release, therefore showing that NO was involved in leptin action. Hence, a short-term treatment with leptin was lipolytic in SD rats as previously demonstrated in Wistar and lean Zucker rats (fa/?) [18], [19], affecting both glycerol and FA release in an NO-dependent manner [18]. Under the same conditions, INF-γ had no effect (Figure 1A, B) whereas this cytokine increased glycerol release on longer treatment times [37], [38].

**Figure 5:**
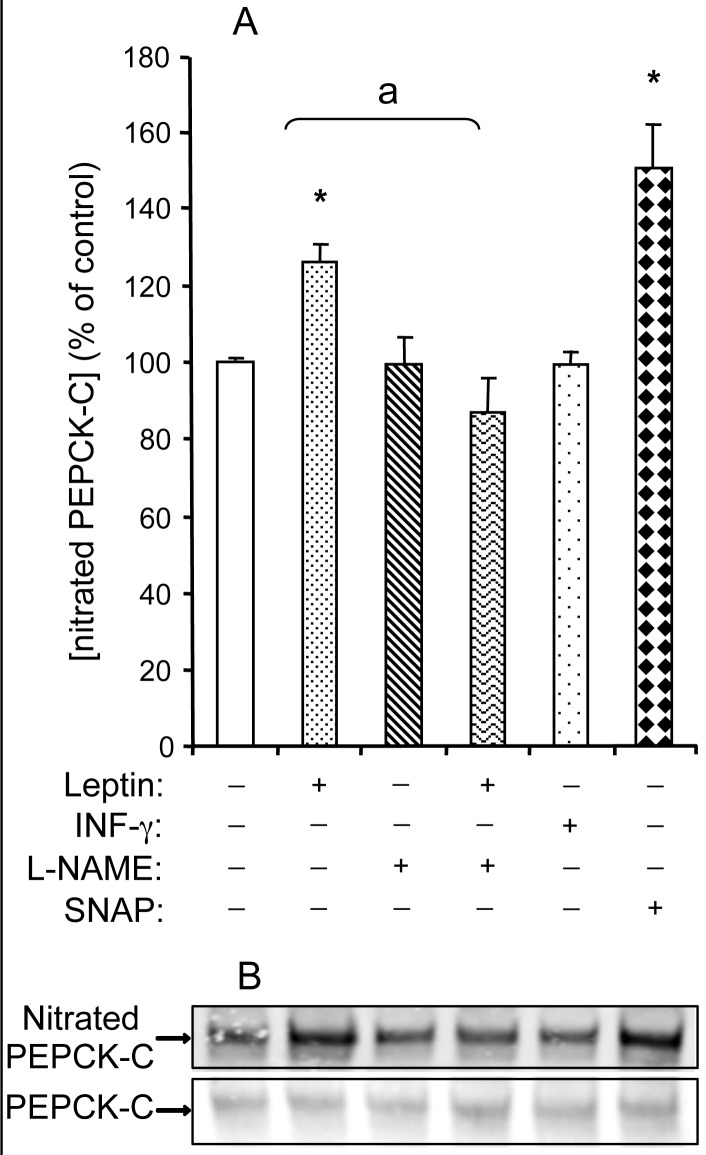
NO-dependent effect of leptin on the nitration of PEPCK-C protein in WAT explants from rats. WAT explants were pre-treated or not with L-NAME (1 mmol/L) for 30 min, then exposed or not to either leptin (10 µg/L), SNAP (1 mmol/L) or IFN-γ (50 µg/L) for 2 h in KRB medium containing 2% BSA. We first immunoprecipited cytosolic proteins using the anti-PEPCK-C antibody then proceeded to western blotting with the anti-nitroprotein antibody. (A) Densitometry scanning in ImageJ software. Each value represents the mean ± SEM, (n = 4) *, *P*<0.01 *vs*. control;^ a^ *P*<0.01 *vs.* leptin-treated explants. (B) Representative autoradiogram.

To check whether leptin would also affect glyceroneogenesis and FA re-esterification, WAT explants were incubated with leptin or INF-γ for 2 h and the incorporation of ^14^C from [1-^14^C] pyruvate into neutral lipids was assessed during the same time length. Leptin induced a 30% decrease in radiolabelled lipids, hence in glyceroneogenesis (Figure 2A). The pre-treatment with L-NAME suppressed leptin action whereas SNAP significantly decreased ^14^C incorporation and IFN-γ had no effect (Figure 2A). AG490, the specific Jak2 inhibitor of leptin receptor, at 10 µmol/L, was inefficient alone but abolished leptin action. Furthermore, leptin reduced glyceroneogenesis in WAT explants from Zucker lean (fa/?) rats with a magnitude similar to that obtained with SD rats and induced the serine phosphorylation of NOS III, activating it (Figure 2B, C). In contrast, no effect of leptin was detected on glyceroneogenesis or NOS III phosphorylation when Zucker obese (fa/fa) rats were used (Figure 2B, C). Therefore, glyceroneogenesis and FA reesterification are depressed by leptin *via* its receptor, in an NO-dependent manner.

### Effect of Leptin and Interferon-gamma on Gene Expression

Since the common knowledge is that glyceroneogenesis relies on PEPCK-C expression and that the amount of this enzyme is directly related to the expression of the gene [13], [14], we evaluated the action that leptin or IFN-γ exerted on PEPCK-C mRNA amount using RT-qPCR. Neither treatment for 2 h with leptin, nor with IFN-γ affected PEPCK-C mRNA (Figure 3). In WAT, NO production depends on the expression of either the inducible NOS II or the constitutive NOS III [24]. The treatment for 2 h with leptin or IFN-γ did not affect the levels of transcripts encoding NOS II or NOS III (Figure 3). We assessed that under our conditions, as expected from previous studies, IFN-γ reduced significantly PEPCK-C mRNA at 8 h of treatment [37], and induced a large 600% increase in NOS II mRNA (data not shown). In contrast, leptin did not change NOS II gene expression, whatever the time of treatment, and decreased PEPCK-C mRNA at 6 h and 18 h [17].

### Effect of Leptin and Interferon-gamma on PEPCK-C Protein Expression and Nitration

Leptin could induce a rapid reduction in the amount of PEPCK-C without affecting gene expression. As shown in figure 4, leptin and SNAP decreased significantly PEPCK-C protein by respectively approximately 30% and 50%. L-NAME reversed leptin effect. Under the same conditions, IFN-γ was without effect.

Therefore, NO acutely decreased PEPCK-C protein without affecting the expression of its gene. Since NO can interact with proteins to form nitrotyrosine derivatives, we studied the action that a 2 h treatment with leptin had on PEPCK-C nitration in WAT explants. We first immunoprecipitated PEPCK-C using a specific antibody then determined the amount of nitrated PEPCK-C by western blot with a selective anti-nitrotyrosine antibody. As presented in figure 5, the 2 h treatment with leptin resulted in a rise in the nitration of PEPCK-C of about 25%, whereas no modification was observed when IFN-γ was used. L-NAME abolished leptin-induced nitration of PEPCK-C whereas SNAP mimicked leptin effect (Figure 5). Hence, leptin, but not IFN-γ induces both the rapid nitration and the decrease in PEPCK-C protein.

## Discussion

Several studies demonstrated that the short exposure of WAT explants or isolated adipocytes from rodents to leptin resulted in lipolytic activity [19], [39], [40] and that NO was implicated in glycerol release. The main findings of the present study are that, on a short-term basis (2 h), leptin decreases WAT glyceroneogenesis and FA re-esterification, therefore resulting in FA release, and that NO mediates this effect. The major glyceroneogenic enzyme, PEPCK-C, is the leptin target. PEPCK-C concentration is rapidly reduced in response to leptin and this decrease is correlated to an increase in the nitration of the protein, which is abolished by the NOS inhibitor L-NAME. Such a short-term leptin action is the consequence of NOS III activation via a protein kinase A-dependent mechanism, in agreement with previous data [25]. As expected also from previous studies, leptin could affect neither NOS III phosphorylation nor glyceroneogenesis when leptin receptor-deficient Zucker rats were used instead of SD rats.

In WAT explants from SD rats, the short exposure to IFN-γ did not change lipolysis. Besides, the expression of *pck1*, NOS II and NOS III genes was not affected by the 2 h-treatment with leptin or IFN-γ. In contrast, IFN-γ alone, or in combination with other cytokines, produces large amounts of NO 6–8 h after treatment, as a result of NOS II induction whereas NOS III expression and activity were either diminished or unchanged [26], [41], [42]. Longer-term treatments with either leptin or IFN-γ reduce glyceroneogenesis and FA re-esterification, therefore producing a rise in FA release [17], [37]. A treatment of 6–18 h with leptin does not change NOS II mRNA but strongly decrease PEPCK-C transcript [17]. Under the same conditions, IFN-γ drastically stimulates NOS II mRNA while down-regulating PEPCK-C mRNA [37]. Hence, both leptin and IFN-γ reduce glyceroneogenesis and PEPCK-C through a NO-dependent process but with different temporal mechanisms: the short-term action of leptin involves NOS III phosphorylation, a process which is actually not affected by IFN-γ, but the action of the latter cytokine is delayed and implicates NOS II induction.

Several post-translational modifications of proteins in response to hormones have been described and among these, tyrosine or serine phosphorylation and lysine acetylation have been widely addressed [4], [16], [43], [44]. More recently, studies demonstrated that NO could react with cysteine (nitrosylation) and tyrosine (nitration) affecting protein stability [28], [29], [31]. NO could interact with PEPCK-C to form either nitrosylated or nitrated derivatives. We decided to focus on variations in the nitrated form of PEPCK-C for several reasons. First, previous discussions of the specificity of the antibodies used to detect nitrosylated or nitrated proteins converged to the notion that those recognizing nitrated proteins are much more reliable than those detecting nitrosylated derivatives [27], [45]. Second, in several pathophysiological disorders, including type 2 Diabetes, an increase in nitrated proteins was detected [27], [31], [46].

It was also shown that nitration compromised the cyclic interconversion between the phosphorylated and unphosphorylated form of tyrosine in proteins [30], [47]. The nitration of tyrosine residues of the insulin receptor beta (IR), of its substrates (IRS1, IRS2), and of phosphoinositide 3-kinase (PI3K/Akt) was observed in the liver of wild-type mice, but not when NOS II KO animals were used [30]. The nitration of key enzymes involved in energy metabolism could modulate their metabolic functions [32] and could be responsible of an insulin-resistant state [30].

As the key enzyme in WAT glyceroneogenesis, PEPCK-C is a target for metabolic disturbances like obesity and type 2 Diabetes [33], [48]. A decrease in FA output participates in the development of obesity. Insulin resistance, which precedes type 2 Diabetes, is linked to a sustained increase in the concentration of blood FA [2]. Glyceroneogenesis and PEPCK-C are rapidly and strongly induced during starvation or during low-carbohydrate, high-fat or high-protein diets [49]. Beta-agonists, retinoic acids, anti-diabetic thiazolidinediones and polyunsaturated FAs, particularly the omega-3 docosahexaenoic acid, increase PEPCK-C gene expression in WAT, isolated explants and adipocytes, in a direct manner [11], [50], [51]. In all these cases, the augmented activity of PEPCK-C is the result of a modification of its quantity that mainly arises from the increase in transcription of its gene and/or stabilization of its mRNA. In contrast, negative actions of hormones or nutrients on PEPCK-C expression are not always at the level of gene regulation. For instance, glucocorticoids exert a negative transcriptional action on the PEPCK-C gene in WAT but glucose rapidly reduces PEPCK-C amount through stimulation of the acetylation of this protein in the liver [15], [16], [52]. Leptin treatment of WAT, whether on a long-term or short-term basis, affects negatively glyceroneogenesis and PEPCK-C levels, thereby limiting FA storage. This action could be one of the beneficial role of leptin in the obese, in which hyperleptinemia is observed. In this context, PEPCK-C nitration is a pertinent mechanism by which leptin could reduce energy uptake and improve energy expenditure, with beneficial effects in the obese.
